# A data driven approach to urban area delineation using multi source geospatial data

**DOI:** 10.1038/s41598-025-93366-x

**Published:** 2025-03-13

**Authors:** Chenyu Fang, Lin Zhou, Xinyue Gu, Xing Liu, Martin Werner

**Affiliations:** 1 Department of Aerospace and Geodesy, Professorship for Big Geospatial Data Management, Technical University of Munich, 85521 Munich, Germany; 2 School of Public Policy, University of Massachusetts Amherst, Amherst, USA; 3 Department of Land Surveying and Geo-Informatics (LSGI), The Hong Kong Polytechnic University, Hong Kong, China; 4 China Academy of Urban Planning & Design Shenzhen, Shenzhen, 518000 Guangdong China

**Keywords:** OpenStreetMap, Feature Engineering (FE), DBSCAN, Data-Driven City, Sustainability, Environmental impact

## Abstract

This study introduces a data-driven, bottom-up approach to urban delineation, integrating feature engineering with the Density-Based Spatial Clustering of Applications with Noise (DBSCAN) algorithm, which represents a significant improvement in precision and methodology compared to traditional approaches that rely on simplistic OpenStreetMap (OSM) road node data aggregations. By employing a broad array of OSM categories and refining data selection through feature engineering, our research significantly enhances the precision and relevance of urban clustering. Using Bavaria, Germany, as a case study, we demonstrate that feature engineering effectively reduces noise and mitigates common DBSCAN clustering pitfalls by filtering out irrelevant and autocorrelated data. The robustness of the proposed method is validated through a comprehensive assessment involving three key elements: (1) a 5% improvement in average accuracy, (2) optimal clustering selections based on entropy values that eliminate the need for prior knowledge, and (3) validation through nighttime light data and Zipf’s law, where a high p-value of 0.99 confirms a good fit, supporting the power law. This study contributes to urban studies by providing a scalable, replicable model that incorporates advanced data processing techniques and multidimensional data sources, supporting improved urban planning and policy-making while effectively delineating urban areas in varied settings.

## Introduction

In the wake of rapid global urbanization, our understanding of urban spaces has evolved significantly. We are currently experiencing a transformation that is reshaping cities across the world, with predictions indicating that by 2050, the global urban population will surge by 2.5 billion, increasing the urbanization rate to 68%^1^. The rapid pace of urbanization presents a critical challenge in defining cities accurately. The way urban areas are delineated directly influences the statistics used in urban analysis, which in turn shapes the conclusions drawn about urban dynamics and development^2^. According to reports from the United Nations Human Settlements Programme (UN-Habitat), achieving the Sustainable Development Goals (SDGs) necessitates addressing key urban challenges, with a foundational requirement being a clear and universally accepted definition of what constitutes a city. Without a precise urban boundary, it becomes difficult to collect reliable data and generate meaningful indicators, which are essential for effective urban planning and sustainable development.

Traditional approaches to urban area delineation, typically based on administrative boundaries or physical metrics such as population density, are increasingly inadequate. These methods fail to capture the timely and heterogeneous nature of urban expansion, leading to discrepancies in urban planning and policy-making^3^. As urban areas continue to evolve, the disparity in regional standards further complicates the objective analysis of urbanization levels^4,4^. In response, there has been a significant shift toward data-driven approaches in urban studies. For example, Ma C. et al. (2024) utilized advanced geospatial data to monitor and capture changes in urban environments, underscoring the importance of integrating diverse data sources to effectively represent the dynamic nature of urban areas^6^. Similarly, Song J. et al. (2023) employed urban big data with an extensive feature set to develop a high-performance map-recovery model for air pollution, highlighting the potential of data-driven approaches in addressing complex urban challenges^7^. These methods leverage diverse data sources to capture dynamic urban transformations more effectively, addressing the need for adaptable and timely urban management solutions.

OpenStreetMap (OSM) represents a transformative development in urban data sources. As a freely available and community-updated platform, OSM offers an unprecedented level of access to comprehensive, real-time geographic data. This global coverage and the up-to-date nature of OSM make it an invaluable resource over traditional data sources, providing detailed insights into urban dynamics without the constraints of cost or outdated information^8^. Despite the advantages of OSM, direct use of its raw data presents challenges, primarily due to issues of data quality and the lack of refined information necessary for detailed urban analysis^9^. While previous studies have utilized OSM for various urban studies, there remains a significant gap in methodologies that effectively harness OSM data through advanced analytical techniques. There is a particular need for comprehensive approaches that integrate feature engineering (FE) and clustering techniques, such as Density-Based Spatial Clustering of Applications with Noise (DBSCAN), to enhance the precision and applicability of OSM data in urban studies^10,10^.

The basis of this study lies in the application of feature engineering and DBSCAN clustering to enhance the use of OSM data for urban area delineation, aiming to develop a robust model that more accurately reflects the complexities of urban environments. By transforming raw OSM data into a format that highlights essential urban characteristics through feature engineering, the research makes the data more actionable for urban studies, allowing for a more natural representation of urban structures without the constraints of predefined cluster counts. Additionally, the use of clustering techniques like DBSCAN, which does not require predefined cluster counts, allows for a more natural and accurate representation of urban structures^12,]^^13,14^.

This paper aims to develop a methodological approach for data-driven city that leverages the power of OSM data to redefine urban area. Specifically, the study seeks to improve the precision of urban area delineation by filtering and clustering relevant OSM data, explore the applicability of DBSCAN clustering in defining urban features and boundaries, and evaluate the performance of the proposed method through validation with external datasets, such as nighttime light data and Zipf’s law. The research question addressed by this study is: how urban areas can be more accurately and dynamically delineated and understood in the context of rapid urbanization and data proliferation.

The primary contributions of this research are manifold: (1) This research employs a multisource big data approach to provide a comprehensive definition of urban areas. (2) From the extensive pool of POI data available in OSM, this study applies feature engineering techniques to selectively extract data points that are most indicative of urban characteristics. (3) The study uses advanced clustering techniques that are not limited by prior knowledge, such as DBSCAN, which allows for a more organic understanding of urban data distributions. (4) To ensure the robustness and scientific reliability of the methodologies developed, this research also validates its findings using other forms of big data.

This paper is organized as follows: Sect.“Related Work” reviews the relevant literature. Section “Data and Methods” describes the data and methods employed in this study. Section “Experiments” details an experiment conducted in Bavaria State, Germany, to test a data-driven city model. Finally, Sects. “Discussion” and 6 discuss the results and provide a conclusion of the study’s findings.

## Related work

### Varying perspectives of delineating urban areas

This section explores the diverse methodologies used to define and identify urban areas, highlighting the evolution from traditional methods to contemporary, data-driven approaches. Traditionally, urban areas have been delineated by administrative boundaries, which are legally defined and provide clear parameters for urban classifications^15,15^. These boundaries, while providing legal clarity, often do not reflect the dynamic nature of urban growth and expansion, which can extend beyond these predefined limits. For example, Song J. & Stettler M. (2022) studied the impact of urban expansion on environmental sustainability, emphasizing the need for dynamic urban spatial delineation methods to better understand and manage the evolving urban environment^17^. The reasons for defining urban areas in this way are not always transparent, leading to differences in urban area delineations that are not comparable across different countries. Furthermore, administrative boundaries often lag behind the current state of urban development, making it challenging for urban planners and policymakers to accurately track and respond to rapid urbanization^18^.

Transitioning to more nuanced methodologies, modern approaches incorporate a variety of metrics such as population density, economic activities, and land use patterns. These data-driven models offer a flexible framework that adapts to the changing realities of urbanization, capturing the complexities of urban sprawl more effectively^19,19^. For example, urban population metrics consider the density and distribution of populations to define urban areas, providing a dynamic parameter that adjusts to demographic changes over time^21,21^. Further, economic activities such as retail distribution and employment density can serve as indicators of urban vitality, while land use patterns, observing changes in land coverage and utilization, reflect urban expansion and the conversion of rural areas into urban settings^23^(Satterthwaite, 2010). Despite the advantages of data-driven models, choosing appropriate thresholds for metrics like population density or economic activity can significantly influence urban delineations, sometimes leading to inconsistencies across different studies or geographic contexts.

Advancements in technology and the availability of large datasets have significantly transformed the delineation of urban areas. Geographic Information Systems (GIS) and remote sensing are pivotal in this transformation, enabling detailed mapping and analysis of urban growth and land use changes^24,24^. The advent of big data analytics complements these technological advances, providing deep insights into urban dynamics through extensive demographic, economic, and environmental data, facilitating more sophisticated urban modeling and decision-making processes^26^. Urban morphology analysis through remote sensing technologies, such as the use of nighttime lighting images or high-resolution satellite data like MODIS and Landsat, has been widely adopted to map urban areas^27,27^. These methods, despite their realism and large-scale applicability, often struggle with issues like cloud cover and varying sensor resolutions, which can obscure the true extent of urban areas^29,29^. Remote sensing, while providing broad coverage and objective data, faces challenges such as the need for frequent calibration and the risk of misclassification, especially in regions where urban and rural features intermingle^31,31^.

An effective method for urban delineation should involve leveraging a comprehensive collection of urban-related big data sources. This integration facilitates the construction and formation of data-driven cities by combining the clear legal authority of administrative definitions with the adaptability and precision of data-driven models. This approach enables a more thorough understanding of urban dynamics, blending historical city contexts with their modern growth patterns, thus supporting urban planners and policymakers in efficiently managing rapid urbanization^18^. This strategic use of diverse data sources enhances the ability to monitor and respond to the evolving landscape of urban environments, ensuring that urban planning and policies are grounded in both current and predictive urban realities.

### Identifying urban areas through OSM data, feature engineering, and DBSCAN

Data-driven approaches to urban area delineation are transforming the way we understand and map urban spaces, adapting to their evolving nature. These methodologies enhance traditional techniques by leveraging diverse data sources, extracting pertinent urban-specific data, and utilizing advanced clustering methods that do not depend on predefined assumptions about urban areas. Reflecting this shift, the UN-Habitat document (2022a) asserts that ‘metropolises are not defined neither by their population, territorial extension nor by the number of their local jurisdictions, but by their functional geography’ (p. 3). This statement underscores the significance of methodologies that enable a dynamic and flexible understanding of urban areas, effectively adapting to their evolving characteristics^33^.

Urban geography has traditionally relied on data from government or proprietary sources, which can be restrictive due to cost, update frequency, and access limitations. OSM, as a freely available and continuously updated repository of global data, presents a significant shift in data sourcing for urban analysis^34,34,34,]^^37,38^. OSM’s comprehensive geographic details offer an alternative that not only enhances coverage but also includes user-generated updates that capture changes in real-time^39^. However, challenges with data quality, which can vary widely in accuracy and detail due to its crowd-sourced nature, necessitate sophisticated validation techniques to ensure the reliability of OSM data for critical urban planning and analysis^7^.

Feature engineering becomes a critical next step in enhancing the use of OSM data for urban analysis. In traditional urban studies, direct use of raw OSM data often fails to capture the nuanced dynamics of urban environments. Feature engineering addresses this by transforming raw data into a refined format that emphasizes the most informative aspects of urban areas^40,40^. The process of feature engineering is typically divided into three key stages: feature selection, feature extraction, and feature construction^42^. Feature selection focuses on dimensionality reduction, aiming to minimize the number of data features while preserving essential information. This step is crucial for simplifying the data analysis process without losing significant insights. Feature extraction transforms a set of features into physically or statistically significant indicators, further refining the data for precise analytical tasks. Lastly, feature construction involves creating new features from existing data through various techniques, including combining different attributes to form new, meaningful indicators. By selecting and engineering features that reflect key urban characteristics, analysts can develop models that more accurately represent urban complexities. This methodological shift is crucial for overcoming the limitations of direct data analysis, which may overlook subtle but critical urban patterns due to noise and irrelevant information^43^.

Building on the foundational work of feature engineering, the transition to clustering methodologies represents a natural progression in identifying urban areas. Traditional clustering techniques often hinge on predetermined assumptions about data structures, such as the number of clusters, which may not accurately reflect the intricate and varied patterns found within urban data. K-means, for example, requires the number of clusters to be specified in advance, and is sensitive to outliers, which can skew the results, especially in the case of urban areas with unevenly distributed data points. Hierarchical clustering, while flexible, suffers from high computational complexity and a tendency to form clusters based on distance metrics that may not align with the true spatial organization of urban features. In contrast, DBSCAN (Density-Based Spatial Clustering of Applications with Noise) offers a robust alternative by clustering data based on local density rather than preset parameters, thereby aligning more closely with the actual spatial distribution of urban features^44,44,44^. This approach is particularly effective in capturing the heterogeneous nature of urban patterns that vary significantly in density and scale, thus providing a more accurate depiction of urban environments.

By synthesizing OSM data, feature engineering, and DBSCAN clustering, this approach provides a robust framework for data-driven urban areas delineation. This integration allows for a comprehensive and nuanced analysis of urban dynamics, supporting urban planners and policymakers with tools that are adaptable to the rapid changes’ characteristic of modern urban environments. The application of these methodologies addresses critical gaps in traditional urban analysis and offers a scalable and replicable system for defining and identifying urban areas.

## Data and methods

This study uses multisource big data to delineate urban areas and analyze urban clustering. The Mutual Information (MI) method is used for feature selection, DBSCAN is applied for clustering, and nighttime light data and Zipf’s law are used to validate the results. These methods enhance the reliability and applicability of the findings in urban studies.

### Data collection

This research integrates four datasets to support a comprehensive urban area analysis: (a) OSM data, (b) Sentinel-2 10 m Land Use/Land Cover data, (c) nighttime light data, and (d) LandScan data, each contributing uniquely to the urban clustering and validation process.

- The OSM data was utilized primarily for its rich categorization of Points of Interest (POIs), which are essential for urban clustering. This dataset, sourced from Geofabrik (https://download.geofabrik.de/) for the year 2021, comprises elements such as nodes, ways, relations, and areas. The study focused on parsing and classifying over 20 million POI entries across 29 predefined categories, as detailed in Table 1, using Python 3.7.0, with further geographical projection into EPSG:25,832—ETRS89 / UTM zone 32N for precise spatial analysis.
- The Sentinel-2 10 m Land Use/Land Cover data, retrieved from https://www.arcgis.com/home/item.html?id=fc92d38533d440078f17678ebc20e8e2, offers detailed land cover types crucial for distinguishing urban from non-urban areas. This 2021 dataset, developed by interpreting ESA Sentinel-2 imagery, categorizes land into nine types: water, trees, flooded vegetation, crops, built areas, bare ground, snow/ice, clouds, and rangeland. The built areas are utilized as ground truth for urban extent in our feature engineering and entropy-based cluster selection, aiding in more accurately delineating urban area.
- Nighttime light data, crucial for validating the accuracy and relevance of our clustering results, was sourced from the Suomi National Polar-orbiting Partnership (Suomi NPP) satellite’s Visible Infrared Imaging Radiometer Suite (VIIRS). This dataset, from June 2021 and available at https://eogdata.mines.edu/products/vnl/, captures artificial lighting indicative of human activities. Extensive processing, including data correction, resampling, and cropping, was conducted to ensure alignment with the urban clusters identified in our study.
- LandScan data, critical for analyzing population distribution within urban clusters identified in our study, was sourced from the Oak Ridge National Laboratory (https://landscan.ornl.gov/).This high-resolution dataset furnishes population density estimates per square kilometer, that are instrumental in verifying how demographic distributions align with the spatial patterns determined through our clustering approach, thereby substantiating the urban delineations.

**Table 1:** Classification and Description of Points of Interest (POI) Types in OpenStreetMap.

Types names of OSM	Description of POI types
aerialway	station, Pylon, Cabin
aeroway	Aerodrome, Runway, Terminal, Apron
amenity	arts center, atm, bank, bar, bench, bicycle parking, bicycle rental, fountain, sustenance, education, transportation, financial, healthcare, public service, facilities, waste management…
barrier	bollard, gate, block, Linear barriers, Access control on highways…
Boundary	Boundary
building	apartments, building, hotel, house, accommodation, commercial, religious, civic/amenity, agricultural/plant production, sports, storage, cars, power/technical buildings, other buildings, additional attributes…
craft	beekeeper, blacksmith, boatbuilder, brewery, carpenter, clockmaker, electronics_repair, embroiderer, goldsmith, handicraft, hvac, jeweller, locksmith, painter, photographer, plumber, pottery, roofer, shoemaker, stonemason, tailor, tiler, watchmaker, winery, wickerwork
emergency	fire hydrant, defibrillator, ambulance station, emergency _ward _entrance, medical rescue, firefighters, lifeguards, assembly point, other structures…
Geological	outcrop, glacier, palaeontological_site, volcano, geothermal, geological_fault
healthcare	alternative, birthing_center, blood_donation, clinic, dentist, doctor, laboratory, midwife, optometrist, pharmacy, physiotherapist, rehabilitation, sample_collection, speech_therapist, vaccination_centre
highway	bus stop, crossing motorway junction, roads, link roads, special road types, paths, sidewalk/crosswalk, cycleway, lifecycle, attributes, other highway features…
historic	memorial, monument…
landuse	commercial, industrial, farmland, forest, meadow, developed land, rural and agricultural land, waterbody…
leisure	park, picnic table, playground, swimming pool…
man made	antenna, flagpole, monitoring station, tower…
Military	airfield, barracks, bunker, checkpoint, danger_area, naval_base, range, training_area
natural	peak, tree, grassland, tree, vegetation, water-related, geology-related…
office	accountant, company, government, adoption _agency…
place	Administratively declared places, Populated settlements, urban, Populated settlements, urban and rural, Other places, Additional attributes
power	cable, catenary_mast, compensator, converter, generator, heliostat, insulator, line, minor_line, plant, pole, portal, substation, switch, tower, transformer, terminal
public transport	platform, station, stop_ area, stop_ position…
railway	station, subway entrance, ventilation shaft, tracks, additional features, stations and stops, other railways…
route	bicycle, bus, canoe, detour, ferry, foot, hiking, horse, light_rail, mtb, pipeline, piste, power, railway, road, running, ski, train, tram
shop	alcohol, antiques, art, books, clothes, convenience, hairdresser, food, mall, charity, health and beauty, do-it-yourself, furniture and interior, electronics, outdoors and sport, stationery
sport	gym, yoga…
telecom	data_center, distribution_point, exchange, manhole, pole, service_device, street_cabinet
tourism	artwork, gallery, hotel, museum…
water	lake, pond, reservoir, river, stream, waterfall, well
waterway	basin, dock, lake, lagoon, oxbow, pond, reservoir, river, riverbank, stream, tidal_channel, waterfall, wetland

### Feature engineering for data processing

Feature engineering is the application of domain knowledge to transform raw data into meaningful features that enhance model performance, reduce complexity, and improve computational speed and accuracy. This process includes identifying the most relevant features, improving data quality through transformation, reducing dimensionality, and creating new features. In the context of this study, feature engineering is crucial for refining the dataset derived from OSM, which includes 29 categories of Points of Interest (POI). Many of these POI categories are not pertinent to urban areas and could adversely affect clustering outcomes. Therefore, feature engineering is employed to select the POI categories most relevant to urban areas.

Feature selection is a key step in this process. It involves ranking features based on their importance and excluding less relevant ones^47^. In this study, we use Mutual Information (MI) to measure the amount of shared information between a feature and the target variable. MI is advantageous because it captures both linear and non-linear relationships between features, making it more robust than methods like Pearson correlation, which only detects linear relationships. This approach helps ensure that only the most relevant features are used in the analysis. The formula for Mutual Information is as follows:

$$I\left(X;Y\right)={\sum }_{x\in X}{\sum }_{y\in Y}P\left(x,y\right){\text{log}}_{2}\frac{P\left(x,y\right)}{P\left(x\right)P\left(y\right)}$$

where P(x,y) is the joint probability mass function of X and Y, and P(x) and P(y) are the marginal probability mass functions of X and Y (Kraskov et al. 2004). Here, the variable Y refers to land-use classes that represent urban areas, which are derived from remote sensing image interpretation. On the other hand, X represents the points clustered from OSM data.

### DBSCAN clustering for defining data-driven urban area

Density-based clustering methods, such as DBSCAN (Density-Based Spatial Clustering of Applications with Noise), excel at identifying clusters by analyzing the density relationships among data points. Unlike other classical clustering algorithms like K-Means or Hierarchical Clustering, which may struggle with arbitrary-shaped clusters or require a predefined number of clusters, DBSCAN is well-suited for handling complex and irregularly spaced data. Given that OSM data often involves noisy, spatially irregular data, and the number and shape of clusters (i.e., urban areas) cannot be predetermined, DBSCAN was selected for its ability to dynamically generate city boundaries without the constraints of other clustering techniques.

DBSCAN, developed by Ester et al. in 1996, operates using two primary parameters: ϵ (the specified radius) and MinPts (minimum points). Understanding these concepts is essential for grasping how DBSCAN works. A core point is one that has at least MinPts other points within a given radius ϵ, satisfying the minimum density requirement for a cluster. These parameters play a crucial role in determining the clustering results. For example, a smaller ϵ value will result in more, smaller clusters, while a larger ϵ will allow more data points to be grouped together into larger clusters. Similarly, increasing the MinPts parameter will ensure that only areas with sufficiently high density are considered part of a cluster.

Unlike traditional clustering methods, DBSCAN can handle noise points (data points that do not belong to any cluster), making it particularly useful when dealing with real-world urban data, which often contains outliers or irrelevant data points. In our study, DBSCAN is used to define city boundaries by clustering Points of Interest (POIs) within OpenStreetMap data. In simpler terms, DBSCAN helps identify regions where urban activity is concentrated by grouping together points that are close to each other and separating out areas where there is little or no urban activity. This ability to handle complex and irregular urban data without requiring predefined cluster numbers is a key advantage of using DBSCAN for city boundary delineation.

**Fig. 1:**
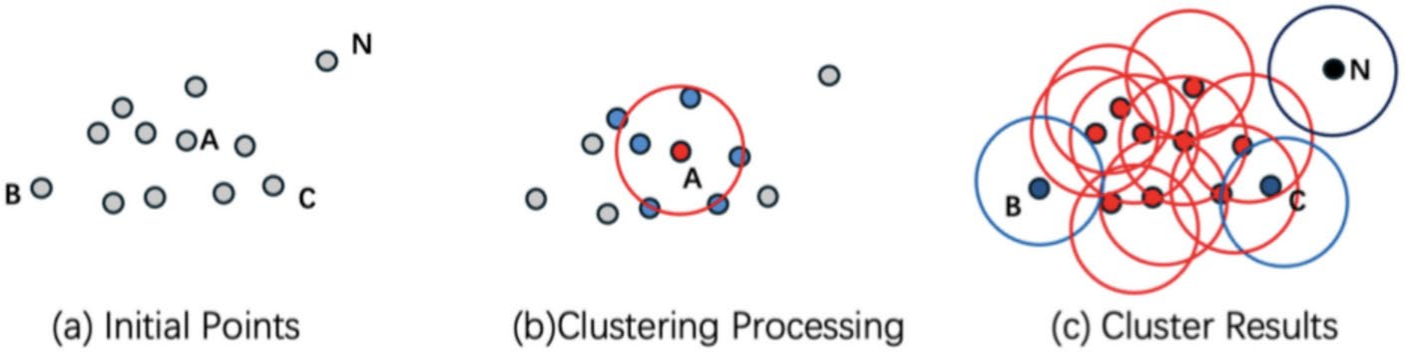
Illustration of DBSCAN Clustering Principles with *MinPts* = 2.

As illustrated in Fig. 1, DBSCAN works by iterating over the dataset, classifying points as core points, border points, or noise points, based on their density relationships. Core points are surrounded by a minimum number of points within the ϵ radius, border points are near core points but do not meet the density threshold themselves, and noise points do not belong to any cluster. By leveraging these relationships, DBSCAN accurately identifies the clusters that correspond to urban areas, while discarding irrelevant points.

### Validating urban clustering: nighttime light data and zipf’s law

In this study, we employ two distinct methodological approaches—nighttime light data and Zipf’s law—to perform a multifaceted validation of our clustering results. These methods are integral to enhancing the reliability and applicability of our findings within the domain of urban studies. The utilization of nighttime light data, which reflects socio-economic activities through the lens of artificial lighting visible from space, combined with the theoretical underpinnings of Zipf’s law that describes city size distribution, provides a comprehensive framework for assessing the accuracy of our clustering methodology.

#### Evaluation using nighttime light data

First, we employ nighttime light data to evaluate the robustness and scientific validity of our proposed clustering methodology. Nighttime light imagery, indicative of human activities and urban development, serves as a robust indicator for socio-economic dynamics, reflecting the intensity of artificial lighting observed via satellite^48^. The procedure begins with the acquisition of nighttime light datasets from NASA’s Earth Data portal (https://www.earthdata.nasa.gov/), followed by meticulous geographic calibration specific to Germany’s contours. We establish a threshold for nighttime light intensity at 3.5^49^, which facilitates the demarcation of urban and non-urban regions. This delineation allows for an empirical comparison between the urban clusters derived from our clustering algorithm and the illuminated areas identified via satellite imagery, thus providing a quantitative measure for validating our clustering results.

#### Application of Zipf’s Law to Urban Studies

Furthermore, we apply Zipf’s law as a theoretical framework for validating the urban clusters identified through our study. It posits that the population size of a city inversely correlates with its rank in the urban hierarchy, suggesting that the largest city is approximately twice as large as the second largest city, three times as large as the third, and so on^50^. This statistical relationship can be leveraged to predict expected urban area sizes and compare them with the clusters identified through our analysis. By comparing the empirical data with the theoretical expectations, we can rigorously evaluate whether our clustering method accurately reflects the underlying urban structure. This comparison not only substantiates the reliability of our clustering outcomes but also aligns our findings with established economic models of urban distribution.

## Experiments

### Background

#### Study area

**Fig. 2:**
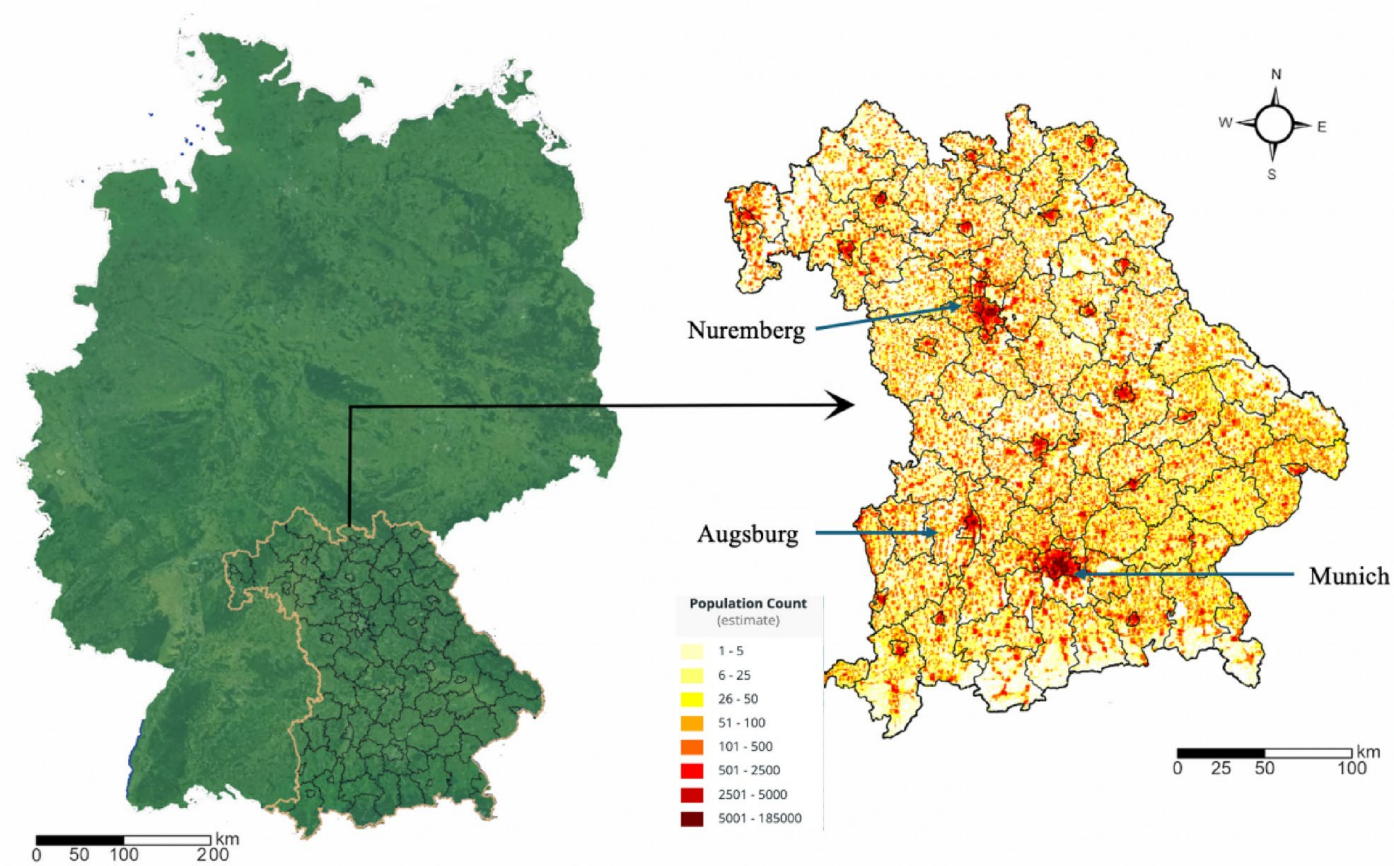
Study Area and Population Map in Bavaria from LandScan.

This study is centered on Bavaria (Fig. 2), located in southern Germany, recognized for its rich geographical diversity, strong economic performance, varied administrative frameworks, and major urban centers. These elements collectively provide valuable insights into urban planning and development, relevant both within the local context and more broadly, potentially influencing policies and practices in similar regions worldwide.

Geographically, Bavaria spans approximately 70,550 square kilometers, characterized by a varied landscape that ranges from the Alpine mountains in the south to expansive plains in the north. This environmental diversity establishes a solid foundation for investigating the effects of ecological and topographical factors on urban development.

Economically, Bavaria is a significant contributor to Germany’s GDP, accounting for about 18.5% with its robust industries and high economic output, as highlighted by a gross regional product of €768.5 billion in 2023 The region’s economic vitality is evident in the bustling activities across both its urban and rural areas, impacting urban expansion and infrastructure development.

Administratively, Bavaria comprises seven regions, 25 municipalities, and 71 counties, each with distinct governance structures and urban planning challenges. This administrative diversity provides a unique lens through which to examine the effects of various governance models on urban development and regional planning.

The major cities of Bavaria, including Munich, Nuremberg, and Augsburg, add further depth to this study. Munich, the state capital, acts as a cultural and economic nucleus with significant global influence and a comprehensive public transportation network. Nuremberg and Augsburg offer contrasting scenarios, showcasing rich historical contexts alongside modern urban challenges, enriching the understanding of urban dynamics in Bavaria.

#### Workflow

**Fig. 3:**
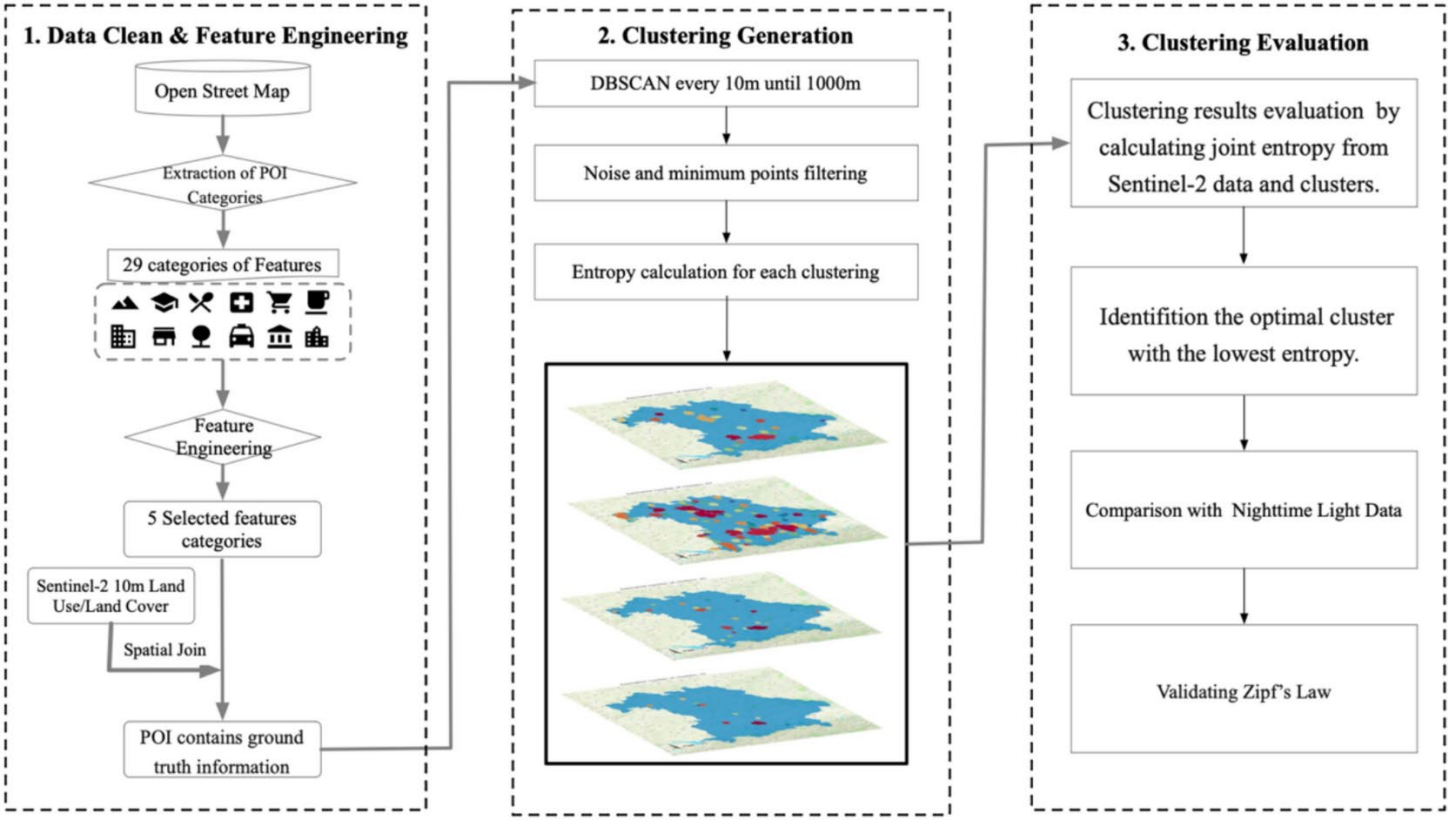
Integrated Workflow for Urban Area Delineation.

The workflow diagram presented in Fig. 3 outlines the methodological framework used in this study for urban area delineation through a three-step process: Data Cleaning & Feature Engineering, Clustering Generation, and Clustering Evaluation.

Step 1: Data Cleaning & Feature Engineering.

The project begins by extracting data from OSM, focusing specifically on Points of Interest (POIs) which are categorized into 29 distinct types. Through feature engineering, this is refined down to the five categories most relevant to urban areas, thereby enhancing the dataset for more targeted analysis. These selected features are then integrated with Sentinel-2 10 m Land Use/Land Cover data, aligning the POIs with actual urban extents. This integration is crucial for accurate geographic data analysis. More specifically, a 10 × 10 m grid is established around each satellite image point, and the POIs are spatially joined to these grids. This arrangement enables precise quantification of each category’s occurrence across the grids. Subsequently, a feature selection algorithm assesses these integrated data points to identify the top 15% with the highest Mutual Information (MI) values for clustering. This process highlights the most significant categories—buildings, amenities, shops, power, and emergency services—out of the original 29, which are critical for accurately representing urban areas. These selected features significantly enhance the clustering process and improve the overall efficacy of data-driven urban planning strategies.

Step 2: Clustering Generation.

Clusters are generated using the DBSCAN algorithm, which methodically iterates through spatial densities (ϵ) from 10 to 1000 m at increments of 10 m, while MinPts starts from 100 to 10,000 and increases in steps of 100 for each iteration. This approach ensures that noise and irrelevant data points are filtered effectively, allowing only sufficiently dense clusters to form. Concurrently, entropy calculations, derived from comparisons between Sentinel-2 10 m Land Use/Land Cover data and the clustering results, are conducted for each cluster to evaluate the precision and coherence of the clustering process. These calculations assist in identifying well-defined urban regions by quantifying the alignment between the clustering outcomes and the ground-truth land-use data.

Step 3: Clustering Evaluation.

In this phase, the clustering results are evaluated by calculating the joint entropy with the ground truth values from Sentinel-2 data to determine the optimal clustering outcomes with the lowest entropy. Minimal entropy indicates the smallest discrepancy between clustering results and the urban areas in Sentinel-2 imagery, suggesting that the clustering outcomes are closely aligned with the built-up area distributions observed in the Sentinel-2 imagery. Further validation is conducted through comparisons with nighttime light data, providing an empirical confirmation of the urban areas identified by the clustering process. Lastly, the clustering results are also validated against Zipf’s Law to ensure that the size distribution of the clusters follows expected theoretical patterns in urban settings.

### Results on classification and analysis

**Fig. 4:**
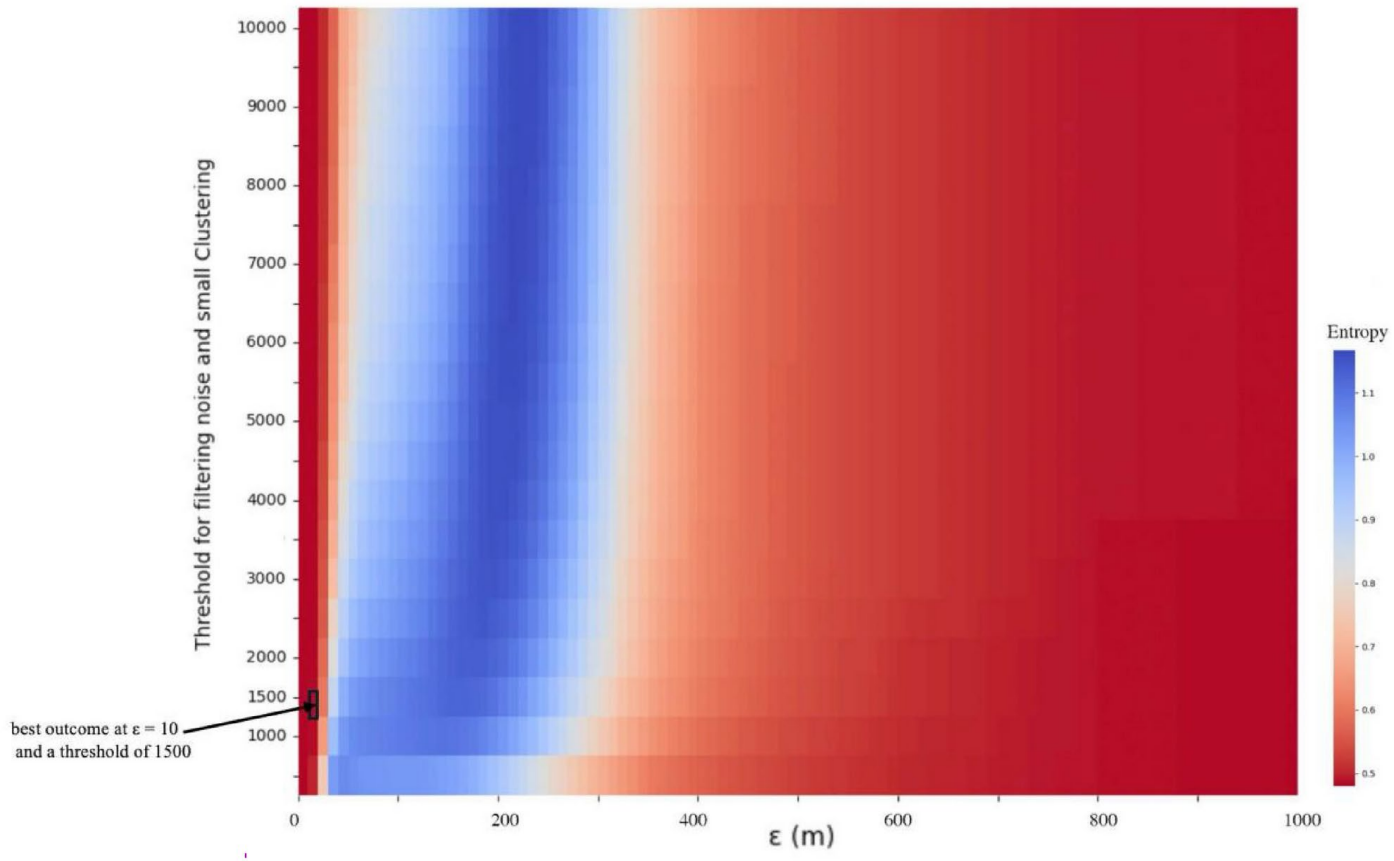
Optimizing Clustering Outcomes Using Minimum Joint Entropy with Sentinel-2 Data.

Figure 4 presents a heatmap representing the traversal results of clustering with ε ranging from 10 to 1000 m and a filtering threshold from 500 to 10,000. The color red indicates lower entropy, while blue signifies higher entropy. In our traversal, the optimal results occur at the bottom left with ε at 10 m and a filtering threshold of 1500. Subsequent analyses will use these parameters to further validate the reliability of the clustering results.

**Fig. 5:**
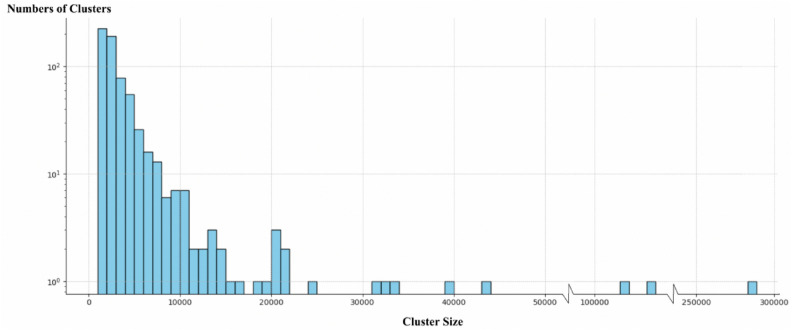
Analysis of Urban Cluster Sizes in Bavaria.

Figure 5 illustrates the distribution of cluster sizes after noise clusters have been filtered out and visualize the spatial dynamics and the scale of urbanization in Bavaria. The results highlight that a total of 652 clusters were identified. Many of these clusters contain fewer than 50,000 points each, indicating a high concentration of smaller, densely packed urban activities or features. Most of these clusters, each containing fewer than 50,000 points, represent a large number of small clusters, highlighting the typical urban distribution in the region—primarily consisting of small to medium-sized clusters. Only three clusters exceed 100,000 points, corresponding to major Bavarian cities: Munich, Nuremberg, and Augsburg, which are among the largest urban centers in the state. The scarcity of large clusters reflects a concentrated urban development in key cities, distinguished by their significant size and comprehensive urban structures. This pattern not only informs about the distribution of urban activity across Bavaria but also about the effectiveness of the chosen DBSCAN parameters in distinguishing between high-density urban cores and less densely populated areas. The declining frequency of larger cluster sizes captured in this figure further suggests that while most urban activities are localized and confined, the significant clusters representing Munich, Nuremberg, and Augsburg highlight central urban hubs with extensive socio-economic activities.

**Fig. 6:**
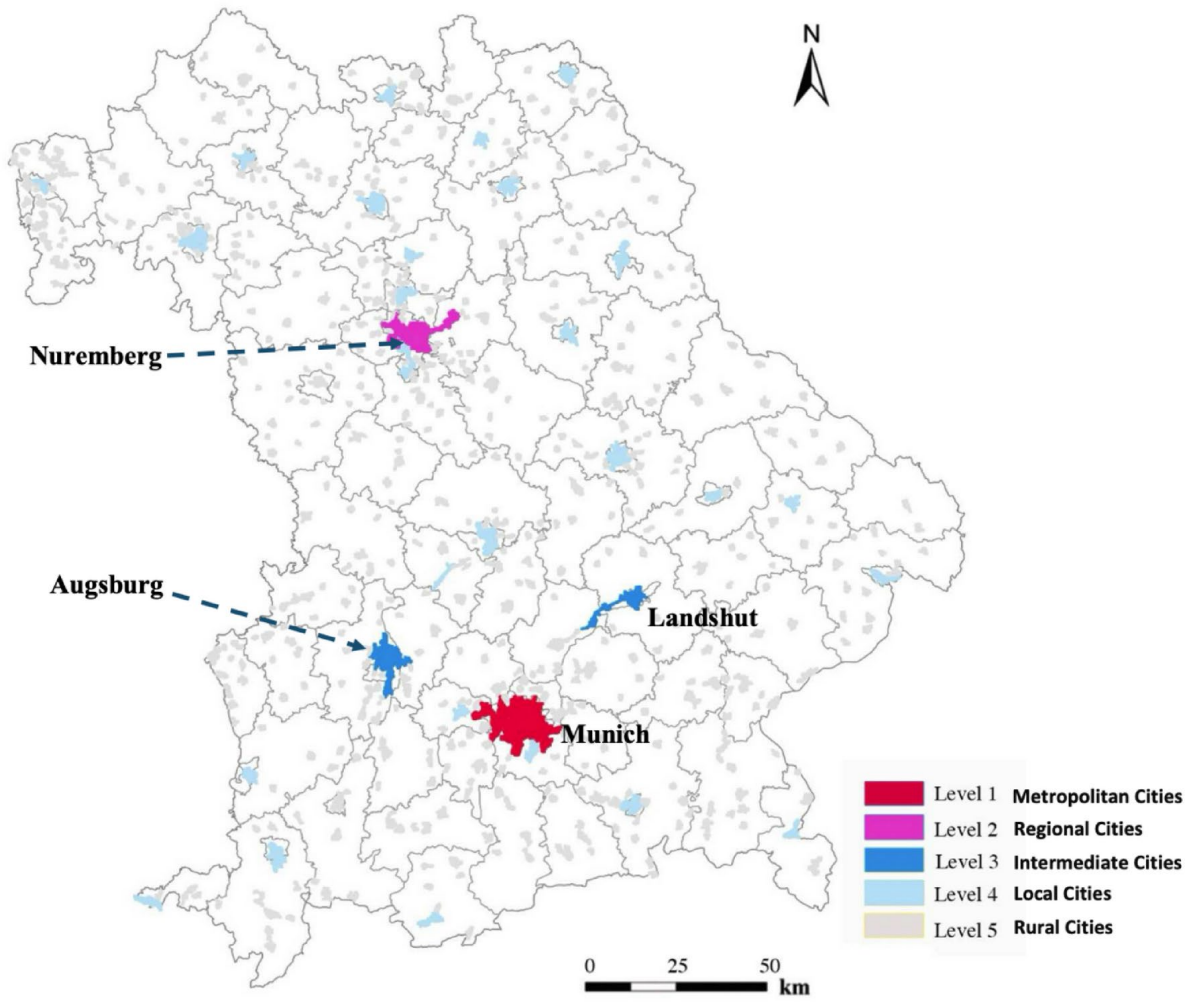
Clustering Results Identifying Urban Areas in Bavaria.

Figure 6 further illustrate the urban clustering in Bavaria, delineating areas into five distinct levels based on natural breakpoints in area data. Table 2 provides a statistical summary including the number of urban clusters (N_urban_), the total area (A_total_) and population (P_total_) of these clusters, alongside the area (A_largest_) and population (P_largest_) of the largest cluster in each level from Level 1 to Level 5.

**Table 2:** Statistics on the urban clusters from Level l to Level 5.

*Cities level*	*N*_*urban*_	*A*_*total*_(km^2^)	*A*_*largest*_ (km^2^)	*P*_*total*_ (people)	*P*_*largest*_ (people)
1 (Metropolitan)	1	5.30 × 10^8^	5.30 × 10^8^	1.80 × 10^6^	1.80 × 10^6^
2 (Regional)	1	3.20 × 10^8^	3.20 × 10^8^	6.50 × 10^5^	6.50 × 10^5^
3 (Intermediate)	2	3.60 × 10^8^	2.10 × 10^8^	5.00 × 10^5^	3.90 × 10^5^
4 (Local)	28	9.60 × 10^8^	7.00 × 10^7^	1.60 × 10^6^	1.30 × 10^5^
5 (Rural)	620	3.10 × 10^9^	1.90 × 10^7^	3.10 × 10^6^	5.20 × 10^4^

Level 1 (Metropolitan Cities) encompasses a single urban cluster representing Munich, Bavaria’s largest city. It covers an expansive area of 530,000 square kilometers and hosts a population of 1.80 million. Traditionally noted for its dense urban core, this cluster stretches into the northeastern and southwestern peripheries, incorporating suburban and peri-urban regions typically absent in standard urban mappings. This expansion reflects Munich’s broad infrastructural and socio-economic influence, extending well beyond its official boundaries to include a more comprehensive metropolitan area.

Level 2 (Regional Cities), represented solely by Nuremberg, spans 320,000 square kilometers with a population of 650,000. The cluster captures a broader urban footprint than typically recognized, indicating areas of influence that extend into neighboring regions. This city stands as a critical economic and historical center, slightly smaller in scale than Munich but equally significant in its regional influence. The urban structure here transitions from the dense metropolitan fabric of Munich to a slightly more dispersed arrangement, characteristic of secondary urban centers.

Level 3 (Intermediate Cities) diversifies with two urban clusters: Augsburg and Landshut, covering a total area of 360,000 square kilometers for 500,000 inhabitants. The clustering suggests an urban expansion that integrates peripheral areas, serving as transitional zones between dense urban settings and suburban areas, thus redefining their urban scope and connectivity.

Level 4 (Local Cities) features 28 clusters covering 960,000 square kilometers and a total population of 1.60 million. Schwabach and Regensburg stand out within this category; Schwabach, with the largest area, and Regensburg, with the highest population, illustrate the shift towards more granular urban spread. The clusters depict a broader urban footprint that encompasses not just the cities themselves but also their extended suburban and semi-rural contexts. This level illustrates the diffusion of urban characteristics into traditionally non-urban areas, blurring the lines between city and countryside.

Level 5 (Rural Towns), the most fragmented, consists of 620 clusters spreading across 3.10 million square kilometers with a corresponding population. Freising and Erding exemplify the largest and most populous clusters, respectively, at this level. These areas represent suburban and peripheral urban forms, where urbanization is less intense but critically important for regional connectivity and development, suggesting these are primarily small communities or peripheral areas that complement the urban landscape.

### Robustness test

#### Evaluation of feature engineering and non-feature engineering results

To validate the efficacy of our clustering approach in urban area delineation, we employed high-resolution Sentinel-2 10 m Land Use/Land Cover imagery as a baseline. Remote sensing image interpretation is a prevalent method for urban detection, which leverages the discernible characteristics of impervious surfaces visible in the imagery to outline urban extents. By using these high-definition images as a ground truth reference, we could critically assess the enhancements brought about by our feature engineering techniques in accurately capturing the urban landscapes.

We adopted the Accuracy (ACC) metric, a widely used statistical measure in classification accuracy assessments, to evaluate the precision of our clustering method after implementing feature engineering. This metric can not only quantify the correctness of our classifications but also help to validate the improvements made by integrating feature engineering into the clustering analysis. ACC is calculated by the formula:

$$ACC=\frac{TP+TN}{TP+TN+FN+FP}$$

where “True Positives” (TP) denote areas where both our clustering method and the remote sensing imagery independently confirm the presence of urban development, and “True Negatives” (TN) refer to areas correctly identified as non-urban by both sources. “False Negatives” (FN) are instances where our clustering fails to detect urban areas that are evident in the imagery, and “False Positives” (FP) occur when our method mistakenly classifies non-urban areas as urban.

**Fig. 7:**
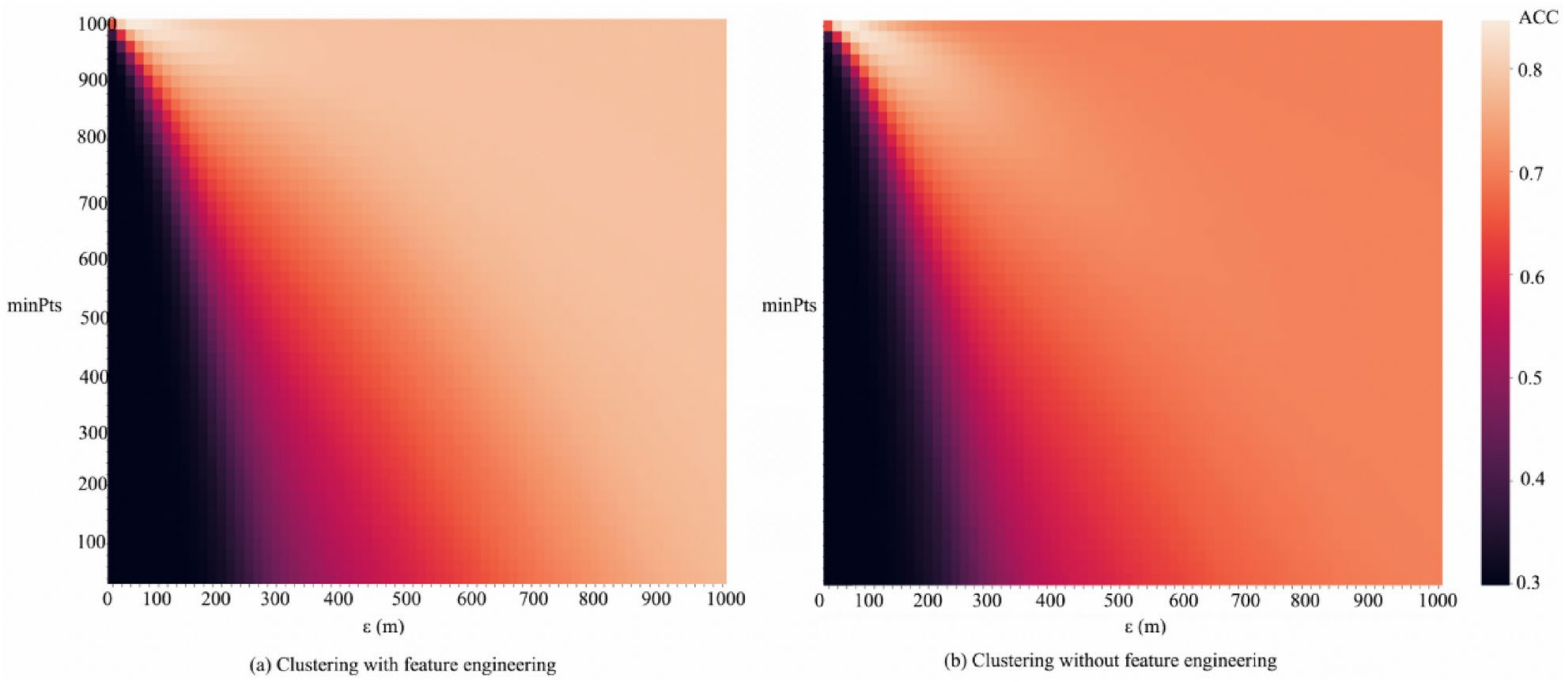
Heatmap Comparison of Clustering Accuracy (ACC). (**a**) shows the clustering results with feature engineering, (**b**) shows the clustering results with feature engineering.

Figure 7 utilizes heatmaps to conduct a comprehensive analysis of clustering accuracy, comparing the outcomes of feature engineering against traditional methods that do not employ feature selection. Figure 7(a) displays the Accuracy (ACC) values for clusters processed through feature engineering, where lighter colors indicate higher ACC values. Conversely, Fig. 7(b) shows ACC values for clusters incorporating all categories of OSM data without the application of feature engineering. The lighter heatmap in Fig. 7(a) reflects the precision gains achieved by filtering out irrelevant or redundant data through feature engineering, which enables more targeted and meaningful clustering outcomes. This approach avoids the noise and overlap seen in non-feature-engineered clusters in Fig. 7(b). By systematically analyzing the effects of ε and MinPts across various configurations, the heatmaps demonstrate that feature engineering consistently improves the accuracy of urban area delineation.

**Fig. 8:**
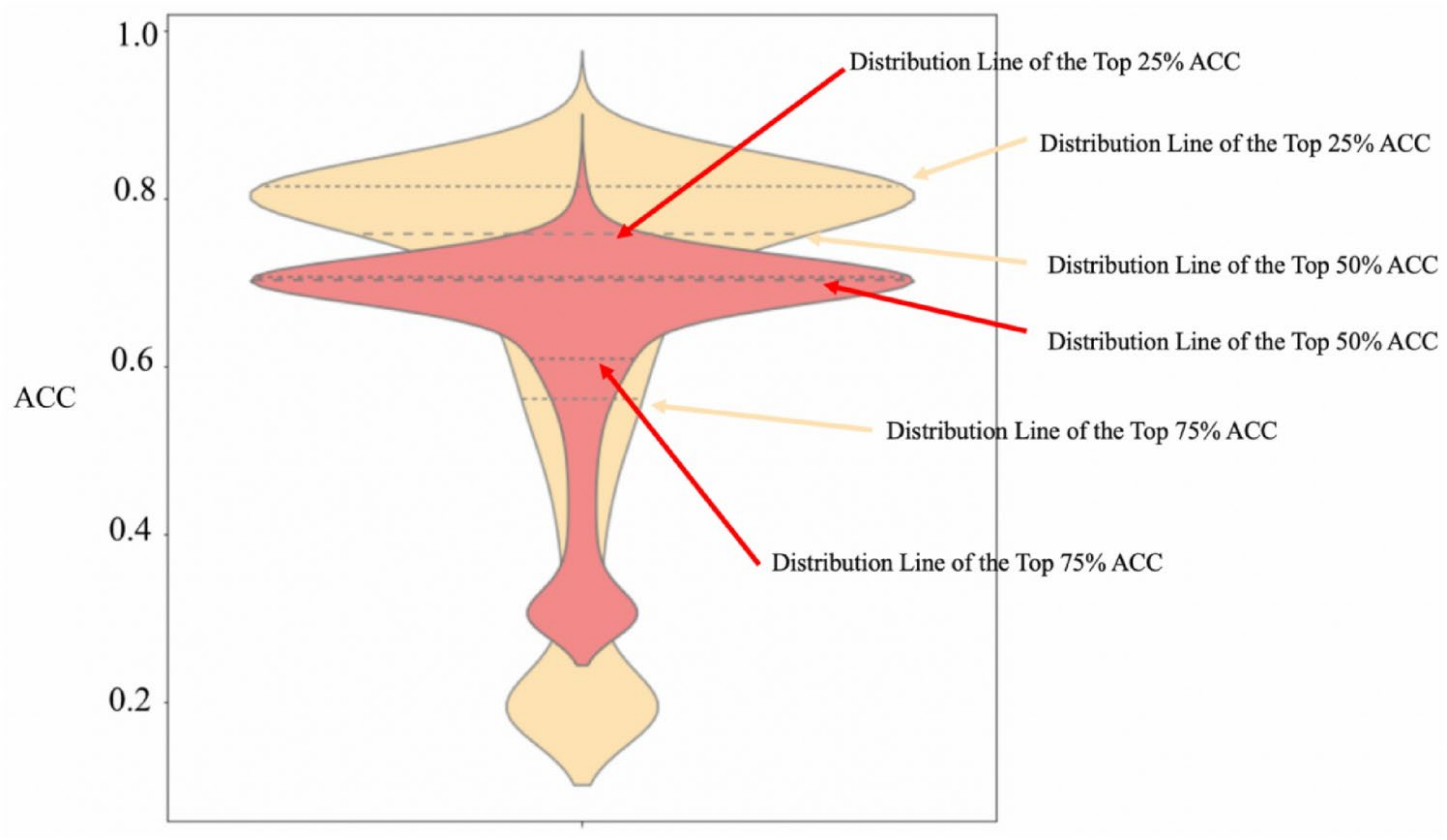
Violin Plots of Clustering Accuracy Comparison between Feature-Engineered and Non-Feature-Engineered Methods.

Figure 8 visually demonstrates the superior performance of feature-engineered clustering compared to traditional non-feature-engineered approaches across a wide range of accuracy metrics. In the heatmap displayed, yellow represents the distribution of Accuracy (ACC) values for clusters analyzed with feature engineering, while red denotes the ACC distribution for clusters processed without the application of feature engineering techniques. The three dashed lines in each plot correspond to the 25th, 50th, and 75th percentile marks of the ACC values, respectively. Notably, the ACC values at both the 25th and 50th percentiles are significantly higher for the feature-engineered outcomes than for the non-engineered ones, indicating a marked improvement in clustering precision. The tail end of the ACC distribution suggests no valid clusters formed due to the stringent criteria of too small a radius (ε) requiring too high a minimum point threshold (MinPts), underscoring a common challenge in parametric clustering methods. Overall, this pattern suggests that feature engineering results in a higher distribution of ACC values, highlighting its efficacy in refining the clustering process.

Table 3 contrasts the average and maximum ACC values between clustering outcomes with and without feature engineering. The average accuracy improved from 0.66 to 0.71, and the maximum accuracy rose from 0.847 to 0.888, indicating an enhancement of 5% and 4%, respectively. This improvement underscores the efficacy of feature engineering in filtering out irrelevant or autocorrelated data, thus reducing noise exposure in DBSCAN clustering and enhancing the overall accuracy of the clustering results.

**Table 3:** Comparison of Clustering Performance with and without Feature Engineering.

*ACC*	*Clustering without FE (%)*	*Clustering with FE*	*Difference (%)*
Avg	71.43	66.32	5.11
Max	88.89	84.74	4.15

#### Entropy-based evaluation of clustering results

During the DBSCAN clustering process, we iterated over the ϵ parameter, ranging from 10 to 1000 m in increments of 10 m, resulting in 100 different clustering outcomes. To determine the best clustering results, we used entropy as a measure to evaluate the clustering quality.

Shannon’s entropy, introduced by Claude Shannon in 1948, measures the uncertainty of an information source. Entropy has since been applied across various fields, including biology, landscape ecology, and urban studies, as a measure of diversity and spatial dispersion . In urban studies, entropy is used to describe land-use patterns, distinguishing between diverse and monofunctional areas^51^, and to define urban areas^39^.

Entropy reaches its maximum when probabilities are evenly distributed and is zero when concentrated in a single location, making it a valuable metric for assessing spatial concentration or dispersion^52^. In the context of urban area definition, employing entropy relies on the assumption that higher entropy values indicate a more accurate resemblance to urban characteristics. Studies by Arcaute et al. (2016)^53^, and Cao et al. (2020)^52^have demonstrated that Shannon’s entropy values strongly correlate with urban areas. Liu et al. (2019)^54^utilized the entropy method to compute optimal threshold values, aiding in the delineation of natural city limits through the aggregation of Voronoi polygons generated from POI service areas. However, this assumption may fail at certain scales or in specific cities due to the inherent complexity of urban environments^49^.

In our study, while we also use entropy to delineate urban areas, we calculate the joint distribution entropy between Sentinel-2 data and clustering data, rather than relying on the assumption that higher entropy values indicate a closer resemblance to urban characteristics. According to the definition of entropy, lower entropy signifies a higher similarity between the two datasets. By incorporating Sentinel-2 data, we extend the application of entropy in urban definition, providing a more nuanced approach to assessing urban areas. The formula for entropy is:

$$H\left(X\right)=-{\sum }_{i=1}^{n}P\left({x}_{i}\right)\text{log}P\left({x}_{i}\right)$$

where P(x_i_) is the probability of occurrence of state x_i_. lower entropy values indicate more similarity and a more accurate representation of urban areas. By minimum entropy, we ensure that the clusters formed reflect the complexity and variability of urban structures, leading to more precise urban areas.

#### Validation of clustering results with nighttime light data

**Fig. 9:**
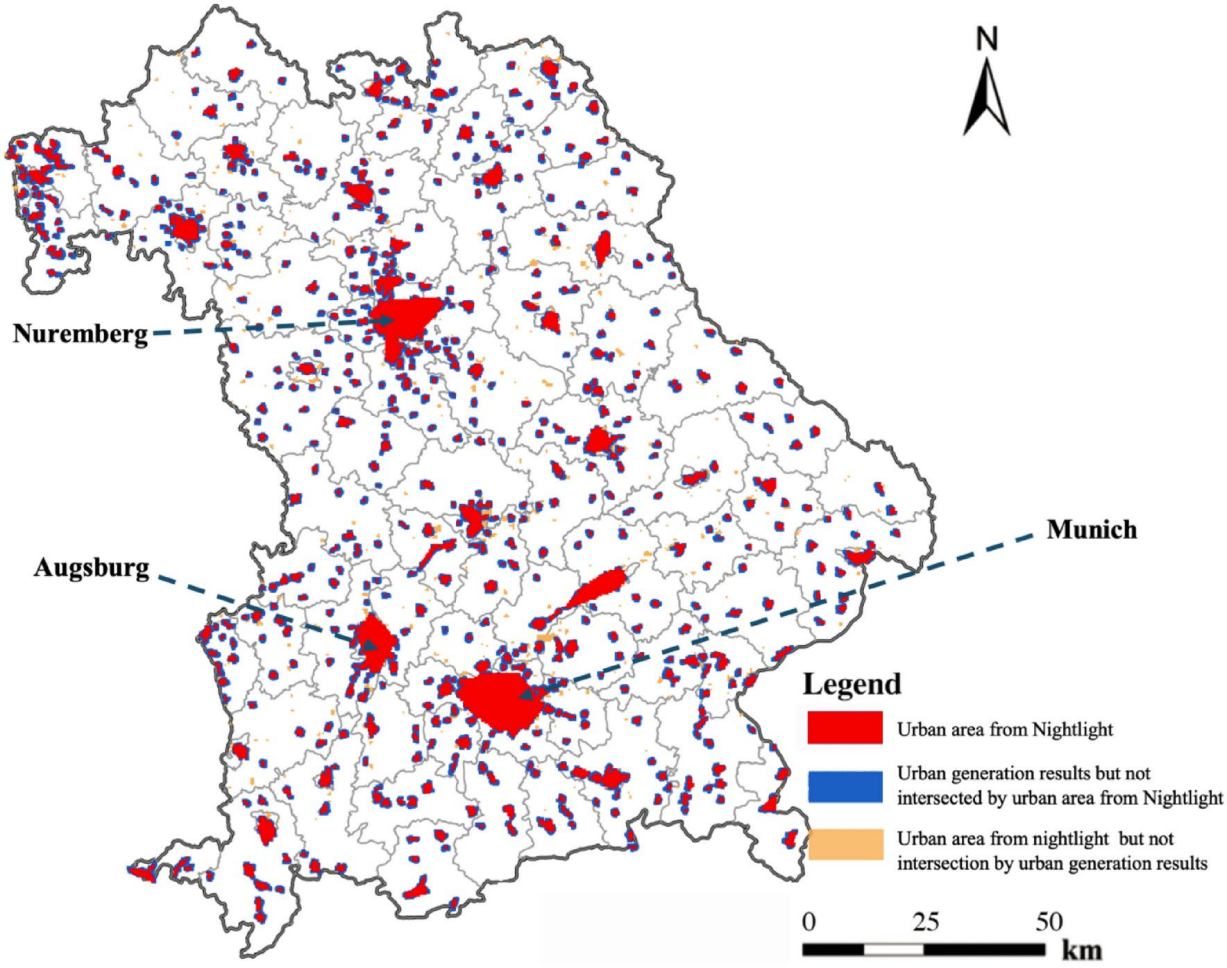
Comparative Analysis of Clustering Results and Nighttime Light Data in Bavaria with 80.6% overlapping area between generated urban area and urban area form Nightlight.

Figure 9 provides a visual comparison of urban clusters identified by our clustering method against urban areas delineated by nighttime light data. Overall, the nighttime light data align well with the urban areas generated by our method, confirming the reliability of both approaches. However, nighttime light data tend to identify smaller, isolated regions as urban areas due to their sensitivity to artificial lighting. In contrast, our clustering-based method imposes stricter criteria, likely reflecting higher requirements for infrastructure and connectivity, thereby producing a more structured and cohesive delineation of urban boundaries. The red areas, highlighted by nighttime light observations, suggest regions of intense human activity and artificial lighting. The significant overlap between these areas and our clusters underscores the efficacy of our method in accurately capturing the true extents of urban regions. Notably, the areas of our clustering results that coincide with nighttime light data comprise 80.3% of the nighttime illumination recorded, underscoring the alignment of our findings with observed urban brightness. It is important to mention that certain small patches of nighttime lights, distant from urban peripheries, are not identified as urban regions, suggesting their exclusion from urban categorization due to their remote locations. Furthermore, the overlap of our clustering results with nighttime light data represents 70.3% of our cluster findings. The areas highlighted in blue signify urban clusters identified by our method that do not align with nighttime light-based urban areas, indicating regions that may be functionally active during the day but less illuminated at night. This discrepancy confirms that our clustering approach may correct some conventional misinterpretations or omissions inherent in the nighttime light methodology. Thus, the yellow areas not covered by our clusters could indicate regions that, while brightly lit, do not exhibit the density or connectivity typically characteristic of urban clusters, such as commercial billboards along highways or large facilities with extensive lighting. This distinction underscores the intricate aspects of urban clustering, which aims to identify true urban fabric rather than merely areas of light intensity.

#### Zipf’s law validation of clustering results

**Fig. 10:**
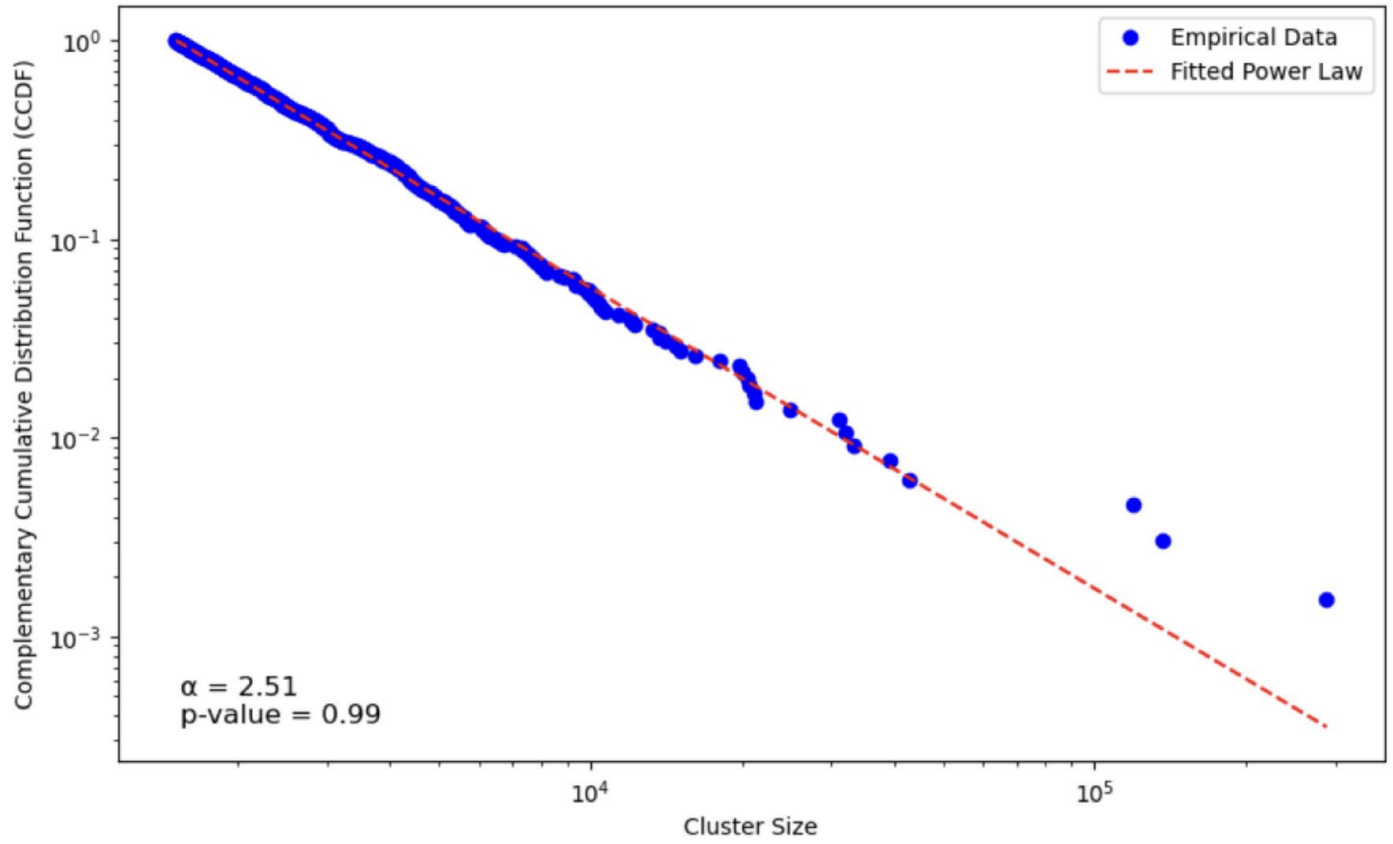
Comparative Analysis of Empirical Clustering Distribution with Theoretical Zipf’s Law in Bavaria.

Figure 10demonstrates the Complementary Cumulative Distribution Function (CCDF) of cluster sizes with a power-law fit, validating urban clustering through Zipf’s law. The graph shows a close alignment between empirical data and the theoretical power-law distribution, with a scaling parameter α of 2.51. This value, higher than the typical urban range of 1 to 2, indicates a “lighter tail” suggesting fewer large cities and a predominance of smaller urban clusters. The significant deviations from the model primarily occur at larger cluster sizes, particularly in major Bavarian cities like Munich, Nuremberg, and Augsburg. This pattern indicates a prevalent occurrence of smaller clusters relative to larger ones, a common feature in urban structures, where numerous small entities exist alongside a few dominant urban hubs^55^.

The high p-value of 0.99 confirms an excellent fit, indicating that the data closely follows a power law, consistent with Zipf’s theory. This suggests that while larger cities are less frequent, they have a significant influence on regional dynamics. This hierarchical pattern supports the effectiveness of the DBSCAN clustering and feature engineering methods used in the study, demonstrating their alignment with established urban development theories. This alignment enhances the credibility of the clustering approach and provides a robust basis for further studies aimed at exploring spatial dynamics and urban growth patterns.

## Discussion

Our study introduces a novel approach to urban area delineation, employing a bottom-up, minimally subjective methodology using OSM data. This technique offers distinct advantages by reducing the reliance on predefined urban definitions, thus enabling a more organic and data-driven formation of urban clusters. In the context of Bavaria, our approach not only identified urban area accurately but also aligned with key theoretical and empirical benchmarks. The validation of our results through nighttime light data confirms their reliability, while adherence to Zipf’s rank-order power law corroborates the scientific robustness of our method. This dual validation underscores the effectiveness of our method in capturing the true scope and scale of urbanization.

While our methodology provides significant advancements in the precision of urban area delineation, the practical implications of these results extend far beyond academic interest. By offering a data-driven, objective approach to defining urban boundaries, our method can be a valuable tool for urban planners and policymakers in several key areas. First, the enhanced accuracy in identifying urban boundaries allows for more effective zoning, land use planning, and resource allocation. For example, clearer delineation of urban areas can help policymakers plan for infrastructure development, such as transportation networks, utilities, and public services, ensuring that urban expansion is managed sustainably. Furthermore, by utilizing OSM data, which is regularly updated and accessible, our method provides a flexible tool for continuously monitoring urban growth, allowing planners to respond more effectively to changing urban dynamics.

Additionally, the improved precision can aid in environmental and social planning by identifying areas at risk of urban sprawl or overpopulation. With more accurate boundaries, urban resilience strategies, such as flood management, green space planning, and disaster preparedness, can be better tailored to actual urban conditions. In this way, the methodological improvements discussed in our study not only contribute to the academic understanding of urbanization but also offer practical solutions for managing urban environments more efficiently and equitably.

Our research significantly advances the field of urban delineation by extending beyond the conventional reliance on specific types of OSM data, such as road nodes, which has been common in many previous studies. For instance, while some approaches focus primarily on clustering road network nodes along with economic units to define urban areas^56,56^, our methodology encompasses a more comprehensive set of OSM data. We implement feature selection to filter out noise and irrelevant data points, which allows for a more detailed and accurate representation of urban environments, akin to how Chen et al. (2022)^58,59^employ spatial indexing to manage data thresholds before clustering, ensuring only pertinent data is analyzed. This allows our method to capture a wider range of urban characteristics, improving the delineation of urban areas compared to traditional methods that rely heavily on road networks or economic units.

Distinguishing our study further is the reduction of reliance on prior knowledge in the clustering process. Utilizing entropy to refine our methodology, we achieve a more autonomous and unbiased urban cluster generation. Compared to methods such as those of Caudillo-Cos et al. 2024, which employ DBSCAN and Shannon’s entropy to define urban boundaries within the Mexican National Urban System but require filtering clusters based on empirical node thresholds, our approach eliminates the need for such filtering by allowing clusters to emerge organically. Similarly, redefine the boundaries of Chinese cities using road node data, with a fixed threshold distance of 300 m determined through prior knowledge. In contrast, our methodology bypasses the necessity of predefined parameters, enabling a bottom-up, unsupervised generation of urban clusters that better captures the complexity and variability of urban patterns. These papers often rely on subjective thresholds applied to data points, whereas our data-driven framework integrates feature engineering and entropy-based cluster refinement to ensure robustness and reproducibility across varying urban contexts. By avoiding subjective thresholds and optimizing the clustering process through entropy-based refinement, our approach ensures higher accuracy and reliability in delineating urban areas, particularly in areas with complex or evolving urban structures.

## Conclusion

This paper introduces a novel method for urban area delineation based on OSM data, applied within Bavaria to analyze urban clustering at various scales. Our methodology is twofold: first, leveraging feature engineering to effectively sift through and select city-related points of interest (POIs) that are most indicative of urban areas; second, utilizing the DBSCAN algorithm to autonomously generate urban structures from the bottom up. Our findings confirm that incorporating feature engineering significantly improves the outcome of clustering algorithms by aligning urban representations more closely with real-world urban patterns.

Despite the progress demonstrated, our study acknowledges several areas for further enhancement and investigation. First, while DBSCAN’s flexibility in not requiring predefined cluster numbers is advantageous, the variability in its hyperparameters introduces an element of uncertainty. Optimizing these settings to balance efficiency with accuracy remains a challenge. Second, as cities expand and regional interconnections strengthen, understanding these relationships becomes crucial. Ignoring the intensity of these connections could lead to misinterpretations about the direction and scale of urban growth. Third, geographical heterogeneity, such as terrain and natural features, plays a critical role in shaping urban landscapes, suggesting that future research should also consider these elements to enhance the accuracy of urban delineation. Fourth, the choices of parameters in feature engineering and DBSCAN clustering, such as the distance threshold and minimum points per cluster, can significantly influence the results, potentially introducing biases in the urban delineation process. While our study explores a range of parameter values to ensure robustness, alternative techniques like k-distance plots, HDBSCAN, or grid search could provide more robust parameter selection and better adapt to varying urban densities, thereby enhancing the reliability and generalizability of the clustering outcomes. Fifth, while nighttime light data and Zipf’s Law are useful for validating urban areas, the former may not always align with actual urban areas, and the latter may not fully capture the complex, decentralized nature of some urban regions. Sixth, while the methodology demonstrates robustness at larger scales, its application to smaller areas, such as cities or neighborhoods, may be constrained by two key factors. On the one hand, the granularity and variability of the available data at smaller scales may not adequately capture the distinctive urban patterns necessary for effective clustering. On the other hand, the weak interconnectivity and high regional similarity often present in smaller-scale urban areas can obscure the delineation of meaningful urban clusters. By addressing these challenges, future research can refine the methodology to provide even more robust tools for urban planners and policymakers engaged in managing and understanding urban environments. In light of the challenges identified, future research could focus on refining the methodology further by incorporating machine learning algorithms for predictive modeling. Integrating advanced machine learning techniques with the current data-driven approach may enhance the ability to predict urban growth patterns, identify emerging urban clusters, and offer real-time insights for urban planners and policymakers. Additionally, expanding the study to include cities with different scales, urban densities, and developmental stages will help validate the broader utility of the approach and refine its parameters for more diverse settings. Moreover, exploring the combination of our urban delineation method with other data sources, such as remote sensing data and social media activity, could improve the accuracy and scalability of urban area definitions. These directions could significantly expand the application and impact of the methodology in dynamic and evolving urban environments.

## Data Availability

Data and code could be found in https://doi.org/10.6084/m9.figshare.28227650.v1.
